# Poly[[μ-aqua-triaqua­[μ_6_-1,3,4,6-tetra­kis­(carboxyl­atometh­yl)-7,8-diphenyl­glycoluril]dizinc] monohydrate]

**DOI:** 10.1107/S1600536811049191

**Published:** 2011-11-25

**Authors:** Chuan-Qiang Li, Wen-Ge Qiu, Hong He, Xiuguang Wang

**Affiliations:** a College of Environmental and Energy Engineering, Beijing University of Technology, Beijing 100124, People’s Republic of China; b College of Chemistry, Tianjin Normal University, Tianjin 300387, People’s Republic of China

## Abstract

In the crystal structure of the title coordination polymer, {[Zn_2_(C_24_H_18_N_4_O_10_)(H_2_O)_4_]·H_2_O}_*n*_, the mol­ecular building block (MBB), *viz* [Zn_2_(CO_2_)_4_(H_2_O)_4_], comprises two Zn^II^ cations, each bridged by three carboxyl­ate groups from different ligand mol­ecules. These two Zn^II^ cations exhibit different coordination environments: a distorted trigonal–pyramidal coordination, as an inter­mediate, is formed by the two coordinated water mol­ecules and three carboxyl­ate groups, and a distorted octa­hedral geometry defined by three water mol­ecules and three carboxyl­ate groups, in which two carboxyl­ate groups from the same side of the clip glycoluril ring and one water mol­ecule are bidentate bridging, whereas others are monodentate units. Every ligand mol­ecule connects four MBBs, thus forming a three-dimensional structure. Extensive intra- and inter­molecular O—H⋯O hydrogen bonding is observed.

## Related literature

For the use of clip ligands in the generation of coordination frameworks, see: Deshpande *et al.* (2008 ▶); Li *et al.* (2008. ▶). For the synthesis of the ligand, see: Kang *et al.* (2004 ▶). 
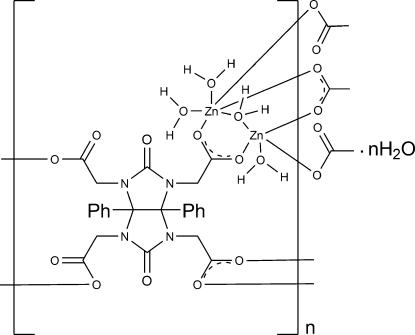


## Experimental

### 

#### Crystal data

[Zn_2_(C_24_H_18_N_4_O_10_)(H_2_O)_4_]·H_2_O
*M* *_r_* = 743.24
Orthorhombic, 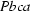

*a* = 18.091 (4) Å
*b* = 15.245 (3) Å
*c* = 19.007 (4) Å
*V* = 5242.0 (18) Å^3^
*Z* = 8
Mo *K*α radiation
μ = 1.92 mm^−1^
*T* = 113 K
0.20 × 0.18 × 0.12 mm

#### Data collection

Rigaku Saturn CCD diffractometer
Absorption correction: multi-scan (*CrystalClear*; Rigaku/MSC, 2005 ▶) *T*_min_ = 0.700, *T*_max_ = 0.802
33644 measured reflections
4616 independent reflections
4022 reflections with *I* > 2σ(*I*)
*R*_int_ = 0.086

#### Refinement

*R*[*F* ^2^ > 2σ(*F* ^2^)] = 0.060
*wR*(*F* ^2^) = 0.151
*S* = 1.13
4616 reflections
413 parameters
3 restraints
H atoms treated by a mixture of independent and constrained refinement
Δρ_max_ = 0.61 e Å^−3^
Δρ_min_ = −0.85 e Å^−3^

### 

Data collection: *CrystalClear* (Rigaku/MSC, 2005 ▶); cell refinement: *CrystalClear*; data reduction: *CrystalClear*; program(s) used to solve structure: *SHELXS97* (Sheldrick, 2008 ▶); program(s) used to refine structure: *SHELXL97* (Sheldrick, 2008 ▶); molecular graphics: *SHELXTL* (Sheldrick, 2008 ▶); software used to prepare material for publication: *SHELXTL*.

## Supplementary Material

Crystal structure: contains datablock(s) global, I. DOI: 10.1107/S1600536811049191/kp2362sup1.cif

Structure factors: contains datablock(s) I. DOI: 10.1107/S1600536811049191/kp2362Isup2.hkl

Additional supplementary materials:  crystallographic information; 3D view; checkCIF report

## Figures and Tables

**Table 1:** Selected bond lengths (Å)

Zn1—O11	2.059 (3)
Zn1—O1	2.063 (3)
Zn1—O4^i^	2.063 (4)
Zn1—O7^ii^	2.091 (3)
Zn1—O12	2.116 (4)
Zn1—O13	2.183 (3)
Zn2—O5^iii^	1.960 (3)
Zn2—O6	2.014 (3)
Zn2—O10^iv^	2.030 (3)
Zn2—O13^v^	2.133 (3)
Zn2—O14	2.183 (4)

Symmetry codes: (i) 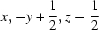; (ii) 
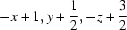
; (iii) 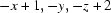; (iv) 
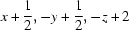
; (v) 
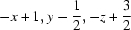
.

**Table 2:** Hydrogen-bond geometry (Å, °)

*D*—H⋯*A*	*D*—H	H⋯*A*	*D*⋯*A*	*D*—H⋯*A*
O15—H15*B*⋯O1^vi^	0.84 (3)	2.54 (6)	3.108 (6)	126 (6)
O15—H15*B*⋯O3^vi^	0.84 (3)	2.29 (6)	2.995 (6)	141 (6)
O15—H15*A*⋯O2^v^	0.83 (3)	2.23 (6)	2.954 (6)	145 (8)
O14—H14*A*⋯O3^iv^	0.85	1.99	2.812 (5)	164
O14—H14*B*⋯O8	0.85	2.30	3.104 (5)	159
O13—H13*B*⋯O2	0.97	1.86	2.684 (5)	142
O13—H13*A*⋯O9^vii^	0.97	1.90	2.671 (5)	134
O12—H12*B*⋯O15^viii^	0.85	1.91	2.723 (6)	158
O12—H12*A*⋯O14^viii^	0.85	2.03	2.827 (5)	156
O11—H11*A*⋯O10^i^	0.85	2.10	2.945 (5)	170
O11—H11*B*⋯O8^i^	0.85	1.98	2.749 (5)	150

Symmetry codes: (i) 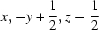; (iv) 
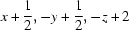
; (v) 
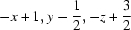
; (vi) 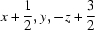; (vii) 
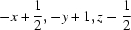
; (viii) 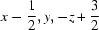.

**Fig. 1.**
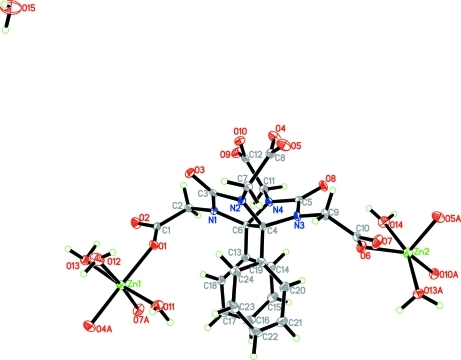
Crystal structure of (I) with the atom labeling and displacement ellipsoids drawn at the 30% probability level. [Symmetry codes: (A) -x + 1/2, -y, z + 1/2; (B) -x, y + 1/2, -z + 1/2; (C) x + 1/2, -y + 1/2, -z.]

**Fig. 2.**
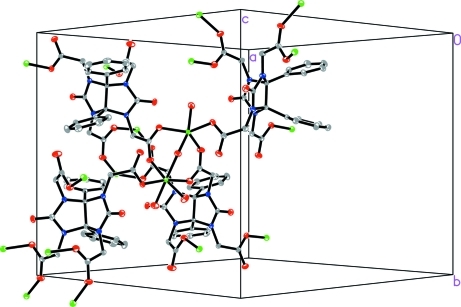
A view of the polymeric structure of (I). The hydrogen atoms and the water molecules in the channels have been omitted for clarity.

## supplementary crystallographic information

### Comment

The clip ligands, such as molecular clips based on concave glycoluril unit or its derivatives, would offer the possibility of the construction of frameworks with novel patterns not easily achievable by planar or linear ligands, which are widely used by chemists in the generation of coordination frameworks (Li *et al.* 2008; Deshpande *et al.* 2008). Herein we chose a multicarboxylate derivative of glycoluril, 1,3,4,6-tetracarboxymethyl-7,8-diphenylglycoluril, as a clip ligand, reacted with zinc nitrate affording a new three-dimensional coordination polymer, (I), {[Zn(II)_2_(*L*)(H_2_O)_4_](H_2_O)}_n_, where *L*=1,3,4,6-tetracarboxymethyl-7,8-diphenylglycoluril.

The molecular building blocks (MBBs) comprise two zinc centres, in which the two Zn atoms are five-coordinated and six-coordinated, respectively (Table 1 and Fig. 1). The different coordination environments in the dinuclear zinc cluster reveal that the Zn1 centre coordinated by five oxygen atoms from three *L* ligands and two water molecules, and Zn2 centre coordinated by six oxygen atoms from three *L* ligands and three water molecules. Each *L* ligand molecule connects four MBBs to form a three-dimensional coordination polymer (Fig. 2). A non-coordinated water molecules occupy the interstitial voids within the framework.

### Experimental

The tetracarboxylic ligand, H_4_L was synthesised according to the literature procedure of Kang (2004). A mixture of H_4_L (20 mg, 0.0380 mmol) and zinc nitrate hexahydrate (28.28 mg, 0.095 mmol) in water (4 mL) was added to a 5 mL sealed glass vial, and heated at 333 K for a week. Colourless rod-like crystals of the title compound were abtained after cooling to room temperature (yield=34% based on H_4_L)

### Refinement

Refinement of F^2^ against ALL reflections. The weighted *R*-factor *wR* and goodness of fit S are based on F^2^, conventional *R*-factors *R* are based on F, with F set to zero for negative F^2^. The threshold expression of F^2^ > 2sigma(F^2^) is used only for calculating *R*-factors(gt) *etc*. and is not relevant to the choice of reflections for refinement. *R*-factors based on F^2^ are statistically about twice as large as those based on F, and R– factors based on ALL data will be even larger.

### Crystal data

**Table:**

[Zn_2_(C_24_H_18_N_4_O_10_)(H_2_O)_4_]·H_2_O	*F*(000) = 3040
*M**_r_* = 743.24	*D*_x_ = 1.884 Mg m^−^^3^
Orthorhombic, *P**b**c**a*	Mo *K*α radiation, λ = 0.71073 Å
Hall symbol: -P 2ac 2ab	Cell parameters from 11423 reflections
*a* = 18.091 (4) Å	θ = 2.4–27.9°
*b* = 15.245 (3) Å	µ = 1.92 mm^−^^1^
*c* = 19.007 (4) Å	*T* = 113 K
*V* = 5242.0 (18) Å^3^	Rod, colourless
*Z* = 8	0.20 × 0.18 × 0.12 mm

### Data collection

**Table:**

Rigaku Saturn 70CCD diffractometer	4616 independent reflections
Radiation source: rotating anode	4022 reflections with *I* > 2σ(*I*)
multilayer	*R*_int_ = 0.086
Detector resolution: 7.31 pixels mm^-1^	θ_max_ = 25.0°, θ_min_ = 2.4°
ω scans	*h* = −21→21
Absorption correction: multi-scan (*CrystalClear*; Rigaku/MSC, 2005)	*k* = −18→18
*T*_min_ = 0.700, *T*_max_ = 0.802	*l* = −14→22
33644 measured reflections	

### Refinement

**Table:**

Refinement on *F*^2^	Secondary atom site location: difference Fourier map
Least-squares matrix: full	Hydrogen site location: inferred from neighbouring sites
*R*[*F*^2^ > 2σ(*F*^2^)] = 0.060	H atoms treated by a mixture of independent and constrained refinement
*wR*(*F*^2^) = 0.151	*w* = 1/[σ^2^(*F*_o_^2^) + (0.0772*P*)^2^ + 11.5013*P*] where *P* = (*F*_o_^2^ + 2*F*_c_^2^)/3
*S* = 1.13	(Δ/σ)_max_ = 0.001
4616 reflections	Δρ_max_ = 0.61 e Å^−^^3^
413 parameters	Δρ_min_ = −0.85 e Å^−^^3^
3 restraints	Extinction correction: *SHELXL97* (Sheldrick, 2008), Fc^*^=kFc[1+0.001xFc^2^λ^3^/sin(2θ)]^-1/4^
Primary atom site location: structure-invariant direct methods	Extinction coefficient: 0.0169 (7)

### Special details

**Table:**

Geometry. All e.s.d.'s (except the e.s.d. in the dihedral angle between two l.s. planes) are estimated using the full covariance matrix. The cell e.s.d.'s are taken into account individually in the estimation of e.s.d.'s in distances, angles and torsion angles; correlations between e.s.d.'s in cell parameters are only used when they are defined by crystal symmetry. An approximate (isotropic) treatment of cell e.s.d.'s is used for estimating e.s.d.'s involving l.s. planes.
Refinement. Refinement of *F*^2^ against ALL reflections. The weighted *R*-factor *wR* and goodness of fit *S* are based on *F*^2^, conventional *R*-factors *R* are based on *F*, with *F* set to zero for negative *F*^2^. The threshold expression of *F*^2^ > σ(*F*^2^) is used only for calculating *R*-factors(gt) *etc*. and is not relevant to the choice of reflections for refinement. *R*-factors based on *F*^2^ are statistically about twice as large as those based on *F*, and *R*- factors based on ALL data will be even larger.

### Fractional atomic coordinates and isotropic or equivalent isotropic displacement parameters (Å^2^)

**Table:**

	*x*	*y*	*z*	*U*_iso_*/*U*_eq_	
Zn1	0.31740 (3)	0.38833 (3)	0.58277 (3)	0.0244 (2)	
Zn2	0.72087 (3)	0.09336 (4)	0.98785 (3)	0.0257 (2)	
O1	0.31600 (17)	0.3726 (2)	0.69057 (16)	0.0236 (7)	
O2	0.2850 (2)	0.5068 (2)	0.72587 (18)	0.0309 (8)	
O3	0.25130 (17)	0.2574 (2)	0.83197 (17)	0.0245 (7)	
O4	0.3152 (2)	0.1190 (2)	0.97441 (18)	0.0328 (9)	
O5	0.3015 (2)	−0.0169 (2)	0.93205 (19)	0.0328 (8)	
O6	0.62798 (18)	0.1136 (2)	0.93178 (19)	0.0286 (8)	
O7	0.59177 (18)	−0.0269 (2)	0.9208 (2)	0.0310 (8)	
O8	0.51124 (19)	0.2312 (2)	1.00804 (16)	0.0279 (8)	
O9	0.3748 (2)	0.4990 (2)	1.0069 (2)	0.0381 (9)	
O10	0.32546 (18)	0.3666 (2)	0.99074 (18)	0.0268 (8)	
O11	0.3804 (2)	0.2759 (2)	0.58136 (19)	0.0363 (9)	
H11B	0.4266	0.2785	0.5737	0.054*	
H11A	0.3657	0.2307	0.5593	0.054*	
O12	0.2201 (2)	0.3113 (3)	0.5791 (2)	0.0416 (10)	
H12A	0.2128	0.2941	0.5371	0.062*	
H12B	0.2266	0.2647	0.6028	0.062*	
O13	0.24415 (19)	0.5017 (2)	0.59018 (17)	0.0272 (7)	
H13A	0.1933	0.4842	0.5818	0.033*	
H13B	0.2473	0.5278	0.6366	0.033*	
O14	0.67873 (19)	0.2110 (2)	1.03965 (19)	0.0342 (8)	
H14B	0.6329	0.2227	1.0421	0.041*	
H14A	0.6971	0.2307	1.0776	0.041*	
N1	0.36573 (19)	0.3234 (2)	0.82313 (18)	0.0166 (8)	
N2	0.3603 (2)	0.1803 (2)	0.84462 (19)	0.0185 (8)	
N3	0.4826 (2)	0.1711 (2)	0.89992 (19)	0.0199 (8)	
N4	0.4545 (2)	0.3105 (2)	0.91913 (18)	0.0184 (8)	
C1	0.3114 (2)	0.4320 (3)	0.7363 (2)	0.0204 (10)	
C2	0.3388 (3)	0.4123 (3)	0.8108 (2)	0.0209 (10)	
H2A	0.3783	0.4530	0.8218	0.025*	
H2B	0.2986	0.4236	0.8434	0.025*	
C3	0.3197 (2)	0.2545 (3)	0.8337 (2)	0.0178 (9)	
C4	0.4399 (2)	0.1956 (3)	0.8382 (2)	0.0172 (9)	
C5	0.4854 (2)	0.2371 (3)	0.9482 (2)	0.0212 (10)	
C6	0.4411 (2)	0.3008 (3)	0.8433 (2)	0.0159 (9)	
C7	0.3268 (3)	0.0946 (3)	0.8513 (2)	0.0212 (10)	
H7A	0.3583	0.0521	0.8279	0.025*	
H7B	0.2798	0.0952	0.8269	0.025*	
C8	0.3140 (2)	0.0645 (3)	0.9261 (2)	0.0218 (10)	
C9	0.4996 (3)	0.0798 (3)	0.9173 (2)	0.0239 (10)	
H9A	0.4768	0.0428	0.8819	0.029*	
H9B	0.4762	0.0663	0.9619	0.029*	
C10	0.5807 (3)	0.0544 (3)	0.9226 (2)	0.0223 (10)	
C11	0.4525 (3)	0.3943 (3)	0.9552 (2)	0.0231 (10)	
H11C	0.4686	0.4393	0.9225	0.028*	
H11D	0.4883	0.3926	0.9932	0.028*	
C12	0.3774 (3)	0.4225 (3)	0.9862 (2)	0.0224 (10)	
C13	0.4999 (2)	0.3428 (3)	0.7982 (2)	0.0191 (9)	
C14	0.5696 (3)	0.3608 (3)	0.8253 (3)	0.0265 (10)	
H14	0.5774	0.3570	0.8735	0.032*	
C15	0.6269 (3)	0.3841 (3)	0.7820 (3)	0.0331 (12)	
H15	0.6731	0.3968	0.8008	0.040*	
C16	0.6152 (3)	0.3886 (3)	0.7088 (3)	0.0353 (13)	
H16	0.6545	0.4012	0.6789	0.042*	
C17	0.5468 (3)	0.3745 (3)	0.6818 (3)	0.0328 (12)	
H17	0.5389	0.3803	0.6337	0.039*	
C18	0.4879 (3)	0.3512 (3)	0.7261 (2)	0.0251 (10)	
H18	0.4411	0.3413	0.7074	0.030*	
C19	0.4733 (3)	0.1581 (3)	0.7713 (2)	0.0203 (9)	
C20	0.5502 (3)	0.1551 (3)	0.7646 (3)	0.0277 (11)	
H20	0.5799	0.1687	0.8031	0.033*	
C21	0.5826 (3)	0.1320 (3)	0.7011 (3)	0.0330 (12)	
H21	0.6338	0.1330	0.6963	0.040*	
C22	0.5384 (3)	0.1072 (3)	0.6447 (3)	0.0354 (13)	
H22	0.5599	0.0916	0.6020	0.042*	
C23	0.4627 (3)	0.1059 (3)	0.6521 (3)	0.0366 (13)	
H23	0.4333	0.0866	0.6151	0.044*	
C24	0.4298 (3)	0.1333 (3)	0.7148 (2)	0.0257 (10)	
H24	0.3785	0.1349	0.7185	0.031*	
O15	0.7371 (3)	0.1916 (3)	0.8159 (3)	0.0617 (13)	
H15A	0.713 (4)	0.150 (4)	0.799 (4)	0.093*	
H15B	0.757 (4)	0.224 (4)	0.786 (3)	0.093*	

### Atomic displacement parameters (Å^2^)

**Table:**

	*U*^11^	*U*^22^	*U*^33^	*U*^12^	*U*^13^	*U*^23^
Zn1	0.0274 (4)	0.0245 (3)	0.0212 (3)	−0.0003 (2)	0.0003 (2)	0.0001 (2)
Zn2	0.0249 (4)	0.0253 (3)	0.0271 (4)	−0.0041 (2)	−0.0018 (2)	0.0009 (2)
O1	0.0285 (18)	0.0260 (16)	0.0163 (16)	0.0054 (14)	−0.0012 (13)	−0.0012 (14)
O2	0.043 (2)	0.0228 (17)	0.0272 (18)	0.0090 (15)	−0.0027 (15)	0.0014 (14)
O3	0.0157 (16)	0.0287 (17)	0.0289 (17)	0.0010 (13)	0.0010 (14)	−0.0010 (14)
O4	0.048 (2)	0.0281 (18)	0.0225 (18)	−0.0025 (16)	0.0010 (16)	0.0011 (15)
O5	0.048 (2)	0.0211 (17)	0.0290 (18)	−0.0078 (16)	0.0056 (17)	0.0021 (15)
O6	0.0238 (18)	0.0242 (17)	0.038 (2)	−0.0003 (14)	−0.0037 (16)	0.0008 (15)
O7	0.0240 (18)	0.0201 (16)	0.049 (2)	0.0055 (14)	−0.0008 (16)	−0.0012 (15)
O8	0.0345 (19)	0.0334 (19)	0.0157 (16)	0.0041 (15)	−0.0071 (14)	0.0027 (14)
O9	0.034 (2)	0.0239 (18)	0.056 (2)	0.0008 (16)	0.0126 (18)	−0.0084 (17)
O10	0.0241 (18)	0.0259 (17)	0.0303 (18)	0.0014 (14)	0.0053 (14)	−0.0036 (15)
O11	0.037 (2)	0.0284 (19)	0.044 (2)	0.0042 (16)	0.0063 (17)	−0.0023 (16)
O12	0.046 (2)	0.046 (2)	0.033 (2)	−0.0173 (19)	0.0009 (17)	−0.0060 (17)
O13	0.0264 (17)	0.0277 (17)	0.0276 (17)	−0.0007 (14)	0.0025 (14)	0.0041 (14)
O14	0.035 (2)	0.0332 (19)	0.035 (2)	−0.0018 (16)	−0.0008 (16)	−0.0056 (16)
N1	0.0154 (18)	0.0180 (17)	0.0164 (18)	0.0013 (14)	−0.0004 (14)	0.0008 (14)
N2	0.0170 (18)	0.0179 (18)	0.0206 (19)	−0.0010 (14)	−0.0007 (15)	0.0038 (15)
N3	0.024 (2)	0.0194 (18)	0.0167 (18)	0.0047 (16)	−0.0055 (15)	0.0016 (15)
N4	0.023 (2)	0.0175 (18)	0.0144 (18)	0.0019 (15)	−0.0023 (15)	−0.0001 (14)
C1	0.016 (2)	0.022 (2)	0.023 (2)	0.0018 (18)	−0.0008 (18)	0.0038 (19)
C2	0.024 (2)	0.021 (2)	0.018 (2)	0.0030 (18)	0.0006 (19)	0.0003 (18)
C3	0.021 (2)	0.022 (2)	0.0096 (19)	−0.0013 (18)	−0.0010 (17)	0.0011 (17)
C4	0.016 (2)	0.018 (2)	0.018 (2)	0.0002 (16)	−0.0008 (17)	0.0041 (17)
C5	0.016 (2)	0.026 (2)	0.021 (2)	0.0006 (18)	0.0044 (18)	0.0013 (19)
C6	0.015 (2)	0.017 (2)	0.016 (2)	0.0012 (16)	−0.0001 (17)	0.0006 (17)
C7	0.022 (2)	0.019 (2)	0.022 (2)	−0.0054 (18)	−0.0019 (19)	0.0009 (18)
C8	0.019 (2)	0.025 (2)	0.022 (2)	−0.0014 (19)	−0.0011 (19)	−0.001 (2)
C9	0.027 (3)	0.020 (2)	0.025 (2)	0.0024 (19)	−0.005 (2)	0.0055 (19)
C10	0.025 (2)	0.023 (2)	0.019 (2)	0.005 (2)	−0.0012 (19)	0.0016 (19)
C11	0.025 (2)	0.024 (2)	0.020 (2)	−0.0045 (19)	−0.003 (2)	−0.0044 (19)
C12	0.029 (3)	0.024 (2)	0.014 (2)	0.004 (2)	−0.0019 (19)	0.0000 (18)
C13	0.019 (2)	0.016 (2)	0.023 (2)	0.0018 (17)	0.0044 (18)	0.0001 (18)
C14	0.025 (3)	0.027 (2)	0.027 (2)	−0.001 (2)	−0.001 (2)	−0.003 (2)
C15	0.021 (3)	0.036 (3)	0.042 (3)	−0.005 (2)	0.002 (2)	−0.002 (2)
C16	0.034 (3)	0.027 (3)	0.045 (3)	0.001 (2)	0.018 (3)	0.008 (2)
C17	0.035 (3)	0.034 (3)	0.030 (3)	0.007 (2)	0.009 (2)	0.010 (2)
C18	0.022 (2)	0.027 (2)	0.026 (2)	0.004 (2)	0.0000 (19)	0.007 (2)
C19	0.027 (2)	0.0133 (19)	0.020 (2)	0.0019 (18)	0.0013 (19)	0.0003 (17)
C20	0.026 (3)	0.027 (2)	0.029 (3)	0.005 (2)	0.001 (2)	−0.003 (2)
C21	0.030 (3)	0.034 (3)	0.035 (3)	0.008 (2)	0.008 (2)	0.000 (2)
C22	0.051 (3)	0.035 (3)	0.020 (3)	0.011 (2)	0.011 (2)	−0.006 (2)
C23	0.050 (3)	0.040 (3)	0.019 (2)	−0.001 (3)	−0.007 (2)	−0.010 (2)
C24	0.030 (3)	0.027 (2)	0.020 (2)	−0.003 (2)	0.000 (2)	−0.001 (2)
O15	0.078 (4)	0.044 (3)	0.063 (3)	−0.014 (2)	0.023 (3)	−0.002 (2)

### Geometric parameters (Å, °)

**Table:**

Zn1—O11	2.059 (3)	N4—C5	1.367 (6)
Zn1—O1	2.063 (3)	N4—C11	1.452 (5)
Zn1—O4^i^	2.063 (4)	N4—C6	1.469 (5)
Zn1—O7^ii^	2.091 (3)	C1—C2	1.529 (6)
Zn1—O12	2.116 (4)	C2—H2A	0.9700
Zn1—O13	2.183 (3)	C2—H2B	0.9700
Zn2—O5^iii^	1.960 (3)	C4—C19	1.519 (6)
Zn2—O6	2.014 (3)	C4—C6	1.607 (6)
Zn2—O10^iv^	2.030 (3)	C6—C13	1.507 (6)
Zn2—O13^v^	2.133 (3)	C7—C8	1.513 (6)
Zn2—O14	2.183 (4)	C7—H7A	0.9700
O1—C1	1.259 (6)	C7—H7B	0.9700
O2—C1	1.252 (5)	C9—C10	1.521 (7)
O3—C3	1.238 (5)	C9—H9A	0.9700
O4—C8	1.238 (6)	C9—H9B	0.9700
O4—Zn1^vi^	2.063 (4)	C11—C12	1.543 (7)
O5—C8	1.266 (6)	C11—H11C	0.9700
O5—Zn2^iii^	1.960 (3)	C11—H11D	0.9700
O6—C10	1.256 (6)	C13—C14	1.389 (6)
O7—C10	1.256 (6)	C13—C18	1.394 (6)
O7—Zn1^v^	2.091 (3)	C14—C15	1.371 (7)
O8—C5	1.233 (5)	C14—H14	0.9300
O9—C12	1.230 (6)	C15—C16	1.408 (8)
O10—C12	1.272 (6)	C15—H15	0.9300
O10—Zn2^vii^	2.030 (3)	C16—C17	1.357 (8)
O11—H11B	0.8500	C16—H16	0.9300
O11—H11A	0.8499	C17—C18	1.404 (7)
O12—H12A	0.8500	C17—H17	0.9300
O12—H12B	0.8501	C18—H18	0.9300
O13—Zn2^ii^	2.133 (3)	C19—C24	1.384 (6)
O13—H13A	0.9700	C19—C20	1.399 (7)
O13—H13B	0.9700	C20—C21	1.388 (7)
O14—H14B	0.8500	C20—H20	0.9300
O14—H14A	0.8500	C21—C22	1.390 (8)
N1—C3	1.357 (5)	C21—H21	0.9300
N1—C6	1.458 (5)	C22—C23	1.377 (8)
N1—C2	1.459 (5)	C22—H22	0.9300
N2—C3	1.365 (6)	C23—C24	1.395 (7)
N2—C7	1.444 (5)	C23—H23	0.9300
N2—C4	1.463 (5)	C24—H24	0.9300
N3—C5	1.363 (6)	O15—H15A	0.83 (3)
N3—C4	1.454 (5)	O15—H15B	0.84 (3)
N3—C9	1.463 (5)		
			
O11—Zn1—O1	85.56 (13)	C19—C4—C6	114.9 (3)
O11—Zn1—O4^i^	87.28 (14)	O8—C5—N3	125.5 (4)
O1—Zn1—O4^i^	170.01 (13)	O8—C5—N4	126.0 (4)
O11—Zn1—O7^ii^	94.52 (14)	N3—C5—N4	108.5 (4)
O1—Zn1—O7^ii^	96.54 (14)	N1—C6—N4	112.9 (3)
O4^i^—Zn1—O7^ii^	90.95 (15)	N1—C6—C13	114.2 (3)
O11—Zn1—O12	89.89 (16)	N4—C6—C13	113.5 (3)
O1—Zn1—O12	87.59 (14)	N1—C6—C4	102.0 (3)
O4^i^—Zn1—O12	85.44 (15)	N4—C6—C4	99.3 (3)
O7^ii^—Zn1—O12	174.17 (15)	C13—C6—C4	113.5 (3)
O11—Zn1—O13	175.16 (13)	N2—C7—C8	114.9 (4)
O1—Zn1—O13	91.19 (12)	N2—C7—H7A	108.5
O4^i^—Zn1—O13	95.49 (13)	C8—C7—H7A	108.5
O7^ii^—Zn1—O13	89.42 (13)	N2—C7—H7B	108.5
O12—Zn1—O13	86.37 (15)	C8—C7—H7B	108.5
O5^iii^—Zn2—O6	109.28 (15)	H7A—C7—H7B	107.5
O5^iii^—Zn2—O10^iv^	102.44 (15)	O4—C8—O5	126.5 (4)
O6—Zn2—O10^iv^	146.94 (14)	O4—C8—C7	119.3 (4)
O5^iii^—Zn2—O13^v^	102.22 (14)	O5—C8—C7	114.1 (4)
O6—Zn2—O13^v^	88.84 (14)	N3—C9—C10	117.4 (4)
O10^iv^—Zn2—O13^v^	93.43 (13)	N3—C9—H9A	107.9
O5^iii^—Zn2—O14	93.81 (14)	C10—C9—H9A	107.9
O6—Zn2—O14	79.72 (13)	N3—C9—H9B	107.9
O10^iv^—Zn2—O14	89.30 (13)	C10—C9—H9B	107.9
O13^v^—Zn2—O14	162.73 (13)	H9A—C9—H9B	107.2
C1—O1—Zn1	127.1 (3)	O7—C10—O6	127.2 (4)
C8—O4—Zn1^vi^	134.7 (3)	O7—C10—C9	113.8 (4)
C8—O5—Zn2^iii^	133.5 (3)	O6—C10—C9	118.9 (4)
C10—O6—Zn2	122.1 (3)	N4—C11—C12	116.6 (4)
C10—O7—Zn1^v^	137.4 (3)	N4—C11—H11C	108.1
C12—O10—Zn2^vii^	120.0 (3)	C12—C11—H11C	108.1
Zn1—O11—H11B	120.6	N4—C11—H11D	108.1
Zn1—O11—H11A	120.6	C12—C11—H11D	108.1
H11B—O11—H11A	105.1	H11C—C11—H11D	107.3
Zn1—O12—H12A	109.4	O9—C12—O10	125.8 (5)
Zn1—O12—H12B	109.4	O9—C12—C11	114.8 (4)
H12A—O12—H12B	105.1	O10—C12—C11	119.3 (4)
Zn2^ii^—O13—Zn1	107.08 (14)	C14—C13—C18	119.1 (4)
Zn2^ii^—O13—H13A	110.3	C14—C13—C6	120.9 (4)
Zn1—O13—H13A	110.3	C18—C13—C6	119.2 (4)
Zn2^ii^—O13—H13B	110.3	C15—C14—C13	121.0 (5)
Zn1—O13—H13B	110.3	C15—C14—H14	119.5
H13A—O13—H13B	108.6	C13—C14—H14	119.5
Zn2—O14—H14B	122.5	C14—C15—C16	119.4 (5)
Zn2—O14—H14A	122.4	C14—C15—H15	120.3
H14B—O14—H14A	105.2	C16—C15—H15	120.3
C3—N1—C6	110.6 (3)	C17—C16—C15	120.2 (5)
C3—N1—C2	122.5 (4)	C17—C16—H16	119.9
C6—N1—C2	125.1 (3)	C15—C16—H16	119.9
C3—N2—C7	122.4 (4)	C16—C17—C18	120.3 (5)
C3—N2—C4	112.6 (3)	C16—C17—H17	119.8
C7—N2—C4	124.3 (3)	C18—C17—H17	119.8
C5—N3—C4	111.9 (3)	C13—C18—C17	119.7 (5)
C5—N3—C9	122.9 (4)	C13—C18—H18	120.1
C4—N3—C9	122.5 (4)	C17—C18—H18	120.1
C5—N4—C11	122.7 (4)	C24—C19—C20	119.1 (4)
C5—N4—C6	112.5 (3)	C24—C19—C4	121.8 (4)
C11—N4—C6	123.2 (3)	C20—C19—C4	118.9 (4)
O2—C1—O1	124.9 (4)	C21—C20—C19	120.5 (5)
O2—C1—C2	116.7 (4)	C21—C20—H20	119.8
O1—C1—C2	118.4 (4)	C19—C20—H20	119.8
N1—C2—C1	116.1 (4)	C20—C21—C22	119.8 (5)
N1—C2—H2A	108.3	C20—C21—H21	120.1
C1—C2—H2A	108.3	C22—C21—H21	120.1
N1—C2—H2B	108.3	C23—C22—C21	119.8 (5)
C1—C2—H2B	108.3	C23—C22—H22	120.1
H2A—C2—H2B	107.4	C21—C22—H22	120.1
O3—C3—N1	125.6 (4)	C22—C23—C24	120.5 (5)
O3—C3—N2	124.9 (4)	C22—C23—H23	119.7
N1—C3—N2	109.5 (4)	C24—C23—H23	119.7
N3—C4—N2	114.5 (3)	C19—C24—C23	120.1 (5)
N3—C4—C19	111.6 (3)	C19—C24—H24	120.0
N2—C4—C19	113.6 (3)	C23—C24—H24	120.0
N3—C4—C6	101.5 (3)	H15A—O15—H15B	114 (4)
N2—C4—C6	99.7 (3)		
			
O11—Zn1—O1—C1	150.5 (4)	N3—C4—C6—N1	−139.5 (3)
O4^i^—Zn1—O1—C1	−165.2 (7)	N2—C4—C6—N1	−21.9 (4)
O7^ii^—Zn1—O1—C1	56.4 (4)	C19—C4—C6—N1	100.0 (4)
O12—Zn1—O1—C1	−119.4 (4)	N3—C4—C6—N4	−23.5 (4)
O13—Zn1—O1—C1	−33.1 (4)	N2—C4—C6—N4	94.1 (3)
O5^iii^—Zn2—O6—C10	−37.3 (4)	C19—C4—C6—N4	−144.1 (4)
O10^iv^—Zn2—O6—C10	159.8 (3)	N3—C4—C6—C13	97.2 (4)
O13^v^—Zn2—O6—C10	65.2 (4)	N2—C4—C6—C13	−145.1 (3)
O14—Zn2—O6—C10	−127.8 (4)	C19—C4—C6—C13	−23.3 (5)
O11—Zn1—O13—Zn2^ii^	−169.0 (16)	C3—N2—C7—C8	−96.5 (5)
O1—Zn1—O13—Zn2^ii^	143.10 (15)	C4—N2—C7—C8	93.1 (5)
O4^i^—Zn1—O13—Zn2^ii^	−44.33 (17)	Zn1^vi^—O4—C8—O5	12.9 (8)
O7^ii^—Zn1—O13—Zn2^ii^	46.57 (16)	Zn1^vi^—O4—C8—C7	−168.7 (3)
O12—Zn1—O13—Zn2^ii^	−129.39 (16)	Zn2^iii^—O5—C8—O4	0.0 (8)
Zn1—O1—C1—O2	22.7 (6)	Zn2^iii^—O5—C8—C7	−178.5 (3)
Zn1—O1—C1—C2	−158.1 (3)	N2—C7—C8—O4	17.2 (6)
C3—N1—C2—C1	−78.8 (5)	N2—C7—C8—O5	−164.2 (4)
C6—N1—C2—C1	117.6 (4)	C5—N3—C9—C10	−79.0 (6)
O2—C1—C2—N1	176.9 (4)	C4—N3—C9—C10	120.9 (4)
O1—C1—C2—N1	−2.4 (6)	Zn1^v^—O7—C10—O6	−9.9 (8)
C6—N1—C3—O3	168.1 (4)	Zn1^v^—O7—C10—C9	174.1 (3)
C2—N1—C3—O3	2.4 (6)	Zn2—O6—C10—O7	−28.8 (6)
C6—N1—C3—N2	−13.5 (5)	Zn2—O6—C10—C9	147.1 (3)
C2—N1—C3—N2	−179.3 (4)	N3—C9—C10—O7	−163.9 (4)
C7—N2—C3—O3	3.9 (6)	N3—C9—C10—O6	19.7 (6)
C4—N2—C3—O3	175.3 (4)	C5—N4—C11—C12	−104.8 (5)
C7—N2—C3—N1	−174.4 (4)	C6—N4—C11—C12	90.9 (5)
C4—N2—C3—N1	−3.0 (5)	Zn2^vii^—O10—C12—O9	15.3 (6)
C5—N3—C4—N2	−86.1 (4)	Zn2^vii^—O10—C12—C11	−167.5 (3)
C9—N3—C4—N2	75.9 (5)	N4—C11—C12—O9	−169.6 (4)
C5—N3—C4—C19	143.1 (4)	N4—C11—C12—O10	12.9 (6)
C9—N3—C4—C19	−54.9 (5)	N1—C6—C13—C14	151.7 (4)
C5—N3—C4—C6	20.2 (4)	N4—C6—C13—C14	20.4 (6)
C9—N3—C4—C6	−177.8 (4)	C4—C6—C13—C14	−92.0 (5)
C3—N2—C4—N3	123.4 (4)	N1—C6—C13—C18	−38.2 (5)
C7—N2—C4—N3	−65.4 (5)	N4—C6—C13—C18	−169.5 (4)
C3—N2—C4—C19	−106.8 (4)	C4—C6—C13—C18	78.1 (5)
C7—N2—C4—C19	64.5 (5)	C18—C13—C14—C15	−1.9 (7)
C3—N2—C4—C6	15.9 (4)	C6—C13—C14—C15	168.2 (4)
C7—N2—C4—C6	−172.8 (4)	C13—C14—C15—C16	−0.9 (7)
C4—N3—C5—O8	172.1 (4)	C14—C15—C16—C17	3.5 (8)
C9—N3—C5—O8	10.2 (7)	C15—C16—C17—C18	−3.1 (7)
C4—N3—C5—N4	−7.7 (5)	C14—C13—C18—C17	2.3 (7)
C9—N3—C5—N4	−169.6 (4)	C6—C13—C18—C17	−168.0 (4)
C11—N4—C5—O8	3.9 (7)	C16—C17—C18—C13	0.3 (7)
C6—N4—C5—O8	169.8 (4)	N3—C4—C19—C24	147.2 (4)
C11—N4—C5—N3	−176.3 (4)	N2—C4—C19—C24	16.0 (6)
C6—N4—C5—N3	−10.4 (5)	C6—C4—C19—C24	−97.9 (5)
C3—N1—C6—N4	−83.2 (4)	N3—C4—C19—C20	−37.9 (5)
C2—N1—C6—N4	82.1 (5)	N2—C4—C19—C20	−169.1 (4)
C3—N1—C6—C13	145.3 (4)	C6—C4—C19—C20	77.0 (5)
C2—N1—C6—C13	−49.4 (5)	C24—C19—C20—C21	3.4 (7)
C3—N1—C6—C4	22.4 (4)	C4—C19—C20—C21	−171.7 (4)
C2—N1—C6—C4	−172.3 (4)	C19—C20—C21—C22	−3.3 (8)
C5—N4—C6—N1	128.8 (4)	C20—C21—C22—C23	0.1 (8)
C11—N4—C6—N1	−65.4 (5)	C21—C22—C23—C24	3.2 (8)
C5—N4—C6—C13	−99.3 (4)	C20—C19—C24—C23	−0.1 (7)
C11—N4—C6—C13	66.5 (5)	C4—C19—C24—C23	174.7 (4)
C5—N4—C6—C4	21.5 (4)	C22—C23—C24—C19	−3.1 (8)
C11—N4—C6—C4	−172.7 (4)		

Symmetry codes: (i) *x*, −*y*+1/2, *z*−1/2; (ii) −*x*+1, *y*+1/2, −*z*+3/2; (iii) −*x*+1, −*y*, −*z*+2; (iv) *x*+1/2, −*y*+1/2, −*z*+2; (v) −*x*+1, *y*−1/2, −*z*+3/2; (vi) *x*, −*y*+1/2, *z*+1/2; (vii) *x*−1/2, −*y*+1/2, −*z*+2.

### Hydrogen-bond geometry (Å, °)

**Table:**

*D*—H···*A*	*D*—H	H···*A*	*D*···*A*	*D*—H···*A*
O15—H15B···O1^viii^	0.84 (3)	2.54 (6)	3.108 (6)	126 (6)
O15—H15B···O3^viii^	0.84 (3)	2.29 (6)	2.995 (6)	141 (6)
O15—H15A···O2^v^	0.83 (3)	2.23 (6)	2.954 (6)	145 (8)
O14—H14A···O3^iv^	0.85	1.99	2.812 (5)	164.
O14—H14B···O8	0.85	2.30	3.104 (5)	159.
O13—H13B···O2	0.97	1.86	2.684 (5)	142.
O13—H13A···O9^ix^	0.97	1.90	2.671 (5)	134.
O12—H12B···O15^x^	0.85	1.91	2.723 (6)	158.
O12—H12A···O14^x^	0.85	2.03	2.827 (5)	156.
O11—H11A···O10^i^	0.85	2.10	2.945 (5)	170.
O11—H11B···O8^i^	0.85	1.98	2.749 (5)	150.

Symmetry codes: (viii) *x*+1/2, *y*, −*z*+3/2; (v) −*x*+1, *y*−1/2, −*z*+3/2; (iv) *x*+1/2, −*y*+1/2, −*z*+2; (ix) −*x*+1/2, −*y*+1, *z*−1/2; (x) *x*−1/2, *y*, −*z*+3/2; (i) *x*, −*y*+1/2, *z*−1/2.

## References

[bb2] Deshpande, M. S., Kumbhar, A. S. & Puranik, V. G. (2008). *Cryst. Growth Des.* **8**, 1952–1960.

[bb3] Kang, J., Ju, H. K. & Jo, J. H. (2004). *Supramol. Chem.* **16**, 175–179.

[bb4] Li, Y., Meng, X., Cao, L., Wang, Y., Yin, G., Gao, M., Wen, L. & Wu, A. (2008). *Cryst. Growth Des.* **8**, 1645–1653.

[bb5] Rigaku/MSC (2005). *CrystalClear* Rigaku/MSC Inc., The Woodlands, Texas, USA.

[bb6] Sheldrick, G. M. (2008). *Acta Cryst.* A**64**, 112–122. 10.1107/S0108767307043930 18156677

